# Assessing the effectiveness of training models for national scale-up of an evidence-based nutrition and physical activity intervention: a group randomized trial

**DOI:** 10.1186/s12889-019-7902-y

**Published:** 2019-11-28

**Authors:** Rebekka M. Lee, Jessica L. Barrett, James G. Daly, Rebecca S. Mozaffarian, Catherine M. Giles, Angie L. Cradock, Steven L. Gortmaker

**Affiliations:** Department of Social and Behavioral Sciences, Prevention Research Center on Nutrition and Physical Activity, Harvard T.H. Chan School of Public Health, 677 Huntington Avenue, 7th Floor, Boston, MA 02115 USA

**Keywords:** Children, Nutrition, Physical activity, Implementation, Training, Afterschool, Obesity prevention, Cost, Out-of-school time

## Abstract

**Background:**

There is a great need to identify implementation strategies to successfully scale-up public health interventions in order to achieve their intended population impact. The Out-of-school Nutrition and Physical Activity group-randomized trial previously demonstrated improvements in children’s vigorous physical activity and the healthfulness of foods and beverages consumed. This implementation study aimed to assess the effects and costs of two training models to scale-up this evidence-based intervention.

**Methods:**

A 3-arm group-randomized trial was conducted to compare effectiveness of in-person and online training models for scaling up the intervention compared to controls. One-third of sites were randomized to the in-person train-the-trainer model: local YMCA facilitators attended a training session and then conducted three learning collaborative meetings and technical assistance. One-third were assigned to the online model, consisting of self-paced monthly learning modules, videos, quizzes, and facilitated discussion boards. Remaining sites served as controls. Fifty-three afterschool sites from three YMCA Associations in different regions of the country completed baseline and follow-up observations using a validated tool of afterschool nutrition and physical activity practices. We used multivariable regression models, accounting for clustering of observations, to assess intervention effects on an aggregate afterschool practice primary outcome, and conducted secondary analyses of nine intervention goals (e.g. serving water). Cost data were collected to determine the resources to implement each training model.

**Results:**

Changes in the primary outcome indicate that, on average, sites in the in-person arm achieved 0.44 additional goals compared to controls (95%CI 0.02, 0.86, *p* = 0.04). Increases in the number of additional goals achieved in sites in the online arm were not significantly greater than control sites (+ 0.28, 95% CI -0.18, 0.73, *p* = 0.24). Goal-specific improvements were observed for increasing water offered in the in-person arm and fruits and vegetables offered in the online arm. The cost per person trained was $678 for the in-person training model and $336 for the on-line training model.

**Conclusions:**

This pilot trial presents promising findings on implementation strategies for scale-up. It validated the in-person training model as an effective approach. The less expensive online training may be a useful option for geographically disbursed sites where in-person training is challenging.

**Trial registration:**

Although this study does not report the results of a health care intervention on human subjects, it is a randomized trial and was therefore retrospectively registered in ClinicalTrials.gov on July 4, 2019 in accordance with the BMC guidelines to ensure the complete publication of all results (NCT04009304).

## Background

Developing and testing strategies for successful scale-up of public health interventions is crucial in order to achieve their potential population impact [1, 2]. The concept of “scale-up” has been defined by the World Health Organization as “deliberate efforts to increase the impact of successfully tested health interventions so as to benefit more people and to foster policy and program development on a lasting basis” [3]. With the prevalence in childhood obesity steadily increasing and racial/ethnic disparities persisting over the past two decades [4–6], identifying novel settings to implement and spread evidence-based interventions (EBIs) that promote nutrition and physical activity is critically important for population health [7]. Out-of-school time (OST) programming reaches 10.2 million children in the United States each year and have the potential to address obesity disparities given that the highest afterschool participation rates are among low income, African-American, and Latino children [7–9].

The Out-of-school Nutrition and Physical Activity (OSNAP) intervention has been rigorously tested in a group-randomized trial and effectiveness has been established. The intervention has demonstrated increases in children’s vigorous physical activity by 36% or an average of 3.2 min [10]. Dietary improvements included increases in consumption of water (1.49 oz./snack) and whole grains (0.10 servings/snack) and decreases in consumption of juice (− 0.61 oz./snack), beverage calories (− 29.1 kcal/snack), foods with *trans* fats (− 0.12 servings/snack), and total calories (− 47.7 kcal/snack) compared to controls [11, 12]. The intervention also increased servings of water at snack and health-promoting policies [13, 14]. OSNAP is a multilevel learning collaborative intervention designed to build the skills and knowledge of afterschool site staff for creating health-promoting policy and practice changes. Over one school year, teams set data-driven action plans around 10 health goals and share experiences creating healthy changes at their sites.

While interventions in out-of-school time, childcare, and school settings have all demonstrated success in promoting healthy behaviors early in life [10, 12, 15–20], little research has rigorously tested strategies for spreading these population-based prevention EBIs for large population reach. In the OST field, experts have established nutrition and physical activity standards [21], but professional development needed for implementation varies widely and no best practices exist for developing this skills of this largely part-time, low-wage workforce [22].

This study is designed to push the field of community-based obesity prevention forward by investigating two implementation strategies for scaling up this EBI [10, 12]: 1) an in-person train-the-trainer approach that develops the capacity of existing YMCA facilitators, and 2) a self-paced online training that can centrally reach a geographically dispersed workforce with greater flexibility. Implementation strategies are considered “methods to enhance the adoption, implementation, sustainment, and scale-up of an innovation” [23]. In the case of this study, the focus is on strategies intended to accelerate scale-up an obesity prevention intervention [10, 12]. By testing these two training modes, we recognize that different strategies are likely needed for broad population reach. In-person train-the trainer approaches have the benefit of increasing skills of local personnel to deliver training, while an online approach allows sites to implement healthy changes if staffing infrastructure is limited or travel and time are a barrier such as in rural and low-resource settings. The train-the-trainer implementation strategy has proven successful for evidence-based practices for improving breast feeding rates [24], increasing motivational interviewing skills [25], and improving nutrition and physical outcomes in childcare settings [26, 27]. The online training implementation strategy builds on the growing evidence for the effectiveness [28, 29] and lower transactional cost [30] of online interactive learning that allows for self-paced formats. Online trainings have grown at rapid pace and led to a substantial shift in education and workforce training in recent years [31]. Meta-analyses and experimental studies have shown similar learning and performance outcomes for online and face-to-face education in the fields of public health and medicine [29, 32]. Notably, a randomized trial comparing in person and online trainings for obesity prevention in childcare settings found similar knowledge change among providers for both training models [33], and childcare managers reported high intention to use Web-based nutrition and physical activity programs [34]. Research on scale-up strategies for population health impact are still evolving, however. Another recent study showed nutrition practice changes after participating in an online training model were promising, but did not demonstrate statistically significant progress compared to controls [35].

By working in partnership with the YMCA—the largest private provider of afterschool programming in the U.S. with worldwide reach to 45 million people in 119 countries—to test models for scale-up, these findings have the potential for real world application and substantial population health impact. The YMCA is led by a national resource office, the YMCA of the USA, which provides centralized leadership, training, and programming and includes staff who played an integral role in recruitment and planning of this study. Across the U.S., there are approximately 2700 independent YMCA Associations across 10,000 communities. Afterschool sites are embedded within these Associations, typically situated physically within YMCA recreation centers and schools.

The aim of this study was to assess the effectiveness and cost of two existing training models (in person and online) for scaling up the OSNAP intervention. Collecting data on implementation cost alongside effectiveness seeks to address questions about the financial investment required as interventions are taken to scale [1, 2]. The primary objective was to determine the impact of each implementation strategy on overall healthy changes in afterschool practices, as measured by a validated observational assessment. The secondary objectives were to determine the impact of each implementation strategy on nine specific healthy practices (e.g. offerings of physical activity, water, fruits and vegetables, and sugary drinks) and assess implementation outcomes such as cost, reach, adoption, and fidelity.

## Methods

### Design and setting

**Fig. 1:**
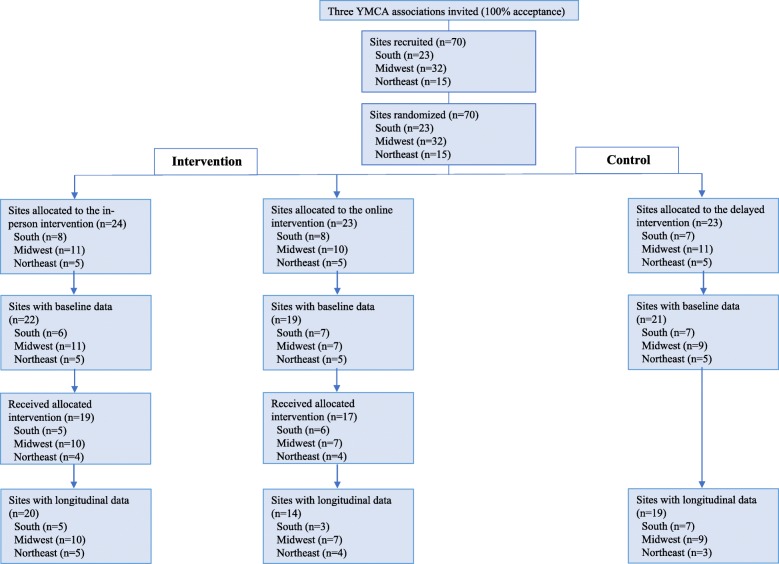
Consort diagram for Out-of-School Time Nutrition & Physical Activity national scale-up trial, 2016–2017

A 3-arm group randomized trial was designed to evaluate the effectiveness of two training models for intervention scale-up (Fig. 1). In Fall 2016, US-based YMCA afterschool sites located in three regions of the US were randomized to either the self-paced online training model, the in person train-the-trainer, or control. The control group was offered training during the 2017–2018 school year (the year after the initial assignment). The primary outcome for this study is an aggregate score of nine healthy nutrition and physical activity practices in afterschool sites. To assess changes in this primary outcome, data were collected at baseline prior to intervention delivery (Fall 2016) and at the end of the school year at post-intervention follow up (Spring 2017). The study was approved by the Harvard T.H. Chan School of Public Health Office of Human Research Administration. An advisory group of YMCA staff, online learning experts, and past OSNAP participants and trainers helped guide the project. The study is guided by the Standards for Reporting Implementation Studies (STaRI) [36] and adheres to CONSORT guidelines; no changes to methods were made after trial commencement.

### Participants and recruitment

The study team collaborated with staff at the YMCA of the USA to identify YMCA Associations that were geographically and racially/ethnically diverse. Eligible Associations were located in one of three regions of the United States (South, Midwest, and Northeast) in communities with less than 75% of residents identified as white in the 2010 U.S. Census. Associations had to have at least 15 afterschool sites and had to demonstrate readiness for implementation and a potential to benefit from the intervention. In this study, eligibility was also determined as having pledged to a commitment to promote nutrition and physical activity as part of the Partnership for a Healthier America by 2016 [37]. Associations that self-reported to YMCA of the USA as having at least one site that met 25–75% of the Healthy Eating and Physical Activity (HEPA) standards [38] in 2016 (i.e. those with >/= 25% standards met readiness criteria, while those with </=75% standards were likely to benefit) were included in the sample. Forty percent of the 450 Associations reporting their achievement of the HEPA standards met this criterion. Once size and demographic criteria were applied, only 12 Associations were eligible. YMCA of the USA staff helped to select three Associations that had limited competing demands and strong leadership. In August 2016, these YMCA of the USA staff invited YMCA leaders from these three eligible Associations to participate via email. All leaders agreed to participate. Subsequently, these Association leaders conducted site recruitment—advertising the initiative to sites with recruitment flyers and emails designed by the study team. Recruitment materials included information on the benefits and goals of OSNAP and site expectations. Seventy sites across the three Associations signed on to participate. Two to three facilitators were recruited at each Association to lead the in-person train-the-trainer arm. One to three adult staff member from each of the study sites were enrolled and participated in human subjects research. These existing site staff completed an implementation survey and practice assessment tool, which measures afterschool site-level data. Each site staff member and facilitator received a $100 gift card for completing fall data collection measures and a second $100 gift card for completing spring data collection measures. The intervention impacts children attending sites via changes to the afterschool environment; however, no data was collected on individual children.

### Random allocation and blinding

Following agreement to participate, sites were randomly assigned to their intervention training status by a researcher outside of the study team using a block randomization procedure of three blocks of sites with random number generation in Microsoft Excel to allow for equal numbers in each study arm as well as geographic variation. No study staff or participants were blinded to randomization. After randomization, YMCA site directors completed a registration form and the principal investigator provided a detailed overview of activities for their assigned OSNAP training group.

### Theoretical basis

**Fig. 2:**
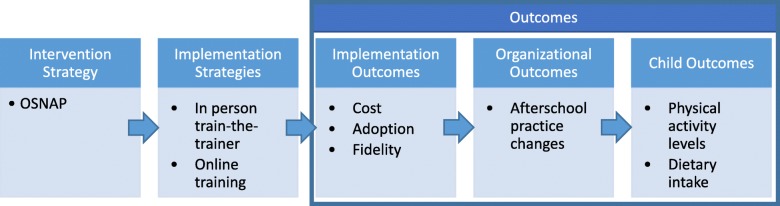
Framework of study intervention, implementation strategies, and outcomes adapted from Proctor’s Conceptual Model of Implementation Research

Proctor's Conceptual Model for Implementation Research serves as the underlying framework for understanding the study intervention, implementation strategies, and outcomes [39]. Fig. 2 depicts how this research tests two implementation strategies (online vs. in person) to deliver the OSNAP evidence-based intervention. Implementation outcomes, such as adoption, implementation, and cost then lead to organizational practice change at the afterschool site (the primary outcome). Child behavioral outcomes, such as physical activity and dietary intake, are not measured in the current study; instead, estimates of the primary outcome are measured via observations of site practices completed by afterschool directors and previously validated against objective measures of children’s physical activity, nutrition, and screen time [40].

### Evidence-based intervention

OSNAP is an environmental change evidence-based intervention (EBI) designed to promote healthy improvements to afterschool practices and policies [10, 12, 13, 41]. The intervention focuses on the following 10 goals: 1) do not serve sugary drinks, 2) do not allow sugary drinks to be brought in during afterschool time, 3) offer water as a drink at snack every day, 4) offer a fruit or vegetable option every day at snack 5) do not serve foods with trans fats, 6) when offering grains, serve whole grains, 7) offer 30 min of physical activity to all children daily, 8) offer 20 min of vigorous physical activity to all children 3 times per week, 9) eliminate use of commercial broadcast and cable television and movies, 10) limit computer and digital device time to homework or instructional only. The intervention is grounded in the social ecological model [42] and social cognitive theory [43]. Over the course of one school year, afterschool site staff build knowledge and skills around each goal and complete action plans to apply changes at their sites. Teams use the Out-of-School Nutrition and Physical Activity Observation Practice Assessment Tool (OSNAP-OPAT) [40] to identify 2–4 goals to focus on site improvement and use decision aids, policy writing guides, and other resources available at www.osnap.org to implement discrete practice, policy, and communication action steps throughout the year. Staff also receive training on the Food & Fun After School curriculum available at www.foodandfun.org. The contact time for full participation in the intervention is estimated to be 10 h over the course of the year and afterschool site staff could earn continuing education units for participating.

### Implementation strategies

Implementation strategies are “methods to enhance the adoption, implementation, sustainment, and scale-up of an innovation” [23]. Two distinct implementation strategies for delivering the OSNAP EBI to afterschool site staff were tested in comparison to a control group. The **in-person train-the-trainer** implementation strategy began with a 6-h training session delivered by the principal investigator at each YMCA Association in fall 2016. Two to three YMCA employees in each region were trained as facilitators to deliver OSNAP locally, gaining skills on OSNAP coordination, facilitation, and content. Facilitators then delivered three 3-h in-person learning collaborative sessions to afterschool staff from sites at their Association (in the fall, winter, and spring of school year 2016–2017) and a 1-h Food and Fun training. They also conducted technical assistance between sessions via site visits, email, text, and phone. The principal investigator conducted two 1-h group coaching calls with trainers between learning collaborative sessions. The **online training** implementation strategy consisted of 7 short, self-paced learning modules to be completed in about 1–1.5 h each month via the Canvas platform [44] between November 2016 and May 2017. It included video clips, interactive quizzes, and a web-based assessment and action planning process. Online discussion boards facilitated by a member of the research team were used to help staff brainstorm, ask questions, and connect with other afterschool site staff. Local facilitators and research staff also provided reminders for participation and logistical support for the platform. Sites in both arms were encouraged to have 2–3 staff members participate in training via email and recruitment flyers. The **control group** did not receive any OSNAP training during the 2016–2017 school year.

## Data collection and measures

### Afterschool child and staff characteristics

A registration form completed by the director at each site in fall 2016 was used to collect descriptive information from sites including the address, enrollment, age range, and racial/ethnic demographics of children served, as well as the age, gender, race/ethnicity, education level, and years of experience of afterschool site staff.

### Primary effectiveness outcomes: afterschool practice changes

The primary outcome for this study is an aggregate score of nine healthy nutrition and physical activity practices in afterschool sites. This outcome was measured with the OSNAP-OPAT—an observational tool completed by afterschool site staff before (fall 2016) and after (spring 2017) the intervention [40]. The tool assesses nine specific health practices aligned with the intervention goals described above: offerings of moderate and vigorous physical activity, screentime, fruits and vegetables, water, juice, whole grains, and sugary drinks from outside the afterschool-provided snacks. The goal of eliminating trans fats is not a focus of this study because it could not be accurately reported in past validation studies and recent policy changes have largely removed trans fats from the food supply. Changes in each of the nine specific practices were assessed as secondary outcomes. The tool was validated during the OSNAP randomized control trial, establishing criterion validity for physical activity and nutrition outcomes with correlations ranging from 0.56 to 0.85 when compared with accelerometry measures of physical activity and direct observation of dietary intake [40]. No changes to the trial outcomes were made after the trial commenced.

### Implementation outcomes

#### Reach

The number of people and sites in the intended audience, in this case afterschool staff and the sites they represent, that participated in the intervention was calculated for each learning collaborative session in the in person model via attendance logs and for each segment in the online model via logins to the Canvas site. Assessing reach allows us to compare the degree to which the intervention was received in each implementation arm.

#### Cost

In order to inform future scale-up, we collected data to compare the costs associated with the two training models as implemented between fall 2016 and spring 2017. We used a societal perspective to determine the costs associated with the in-person and online implementation strategies by accounting for all costs associated with delivering the training implementation strategies [45]. First, we identified activities used in each implementation strategy arm, and then identified resources associated with these activities and valued those resources. Activities included learning collaborative sessions and technical assistance for both strategies, plus train-the-trainer costs for the in-person arm. We estimated costs by measuring the quantity of resources required and their associated costs specific to each Association. The resources we costed in each activity were salaries and time of the facilitators, trainers, site directors, and afterschool site staff engaged in preparation, training, and technical assistance; OSNAP training materials; and travel costs. Salaries were estimated using data from the Bureau of Labor Statistics 2016 metropolitan locations including a 1.4556% fringe rate. Research staff time was self-reported by the three individuals delivering intervention content. Trainer and afterschool site staff time was estimated from attendance sheets, online logins, and technical assistance logs. Travel costs (ground transport, airfare, and lodging), including travel to and from each location from Boston, MA where the research study is based, were gathered from administrative records. Costs were also categorized by payer, contrasting those incurred by the YMCA Associations versus those incurred by the research team. Costs do not include turnover or ongoing training costs over time and do not include additional equipment costs or expenditures as a result of delivering the evidence-based intervention at the afterschool site level (e.g., purchasing of physical activity equipment, changes in costs associated with fruit and vegetable purchases). No costs for research activities (e.g. survey development, entry, analysis) were included. Costing methods are consistent with established literature [45, 46] and with costing protocols used in the CHOICES project [47, 48].

#### Adoption

Adoption is the initial uptake of an innovation. In this study, adoption was operationalized as the goals selected by afterschool site staff on action plans during the 2016–2017 school year. This allowed us to track the types of nutrition and physical activity practices and policies sites focused on as part of the OSNAP intervention. Afterschool site staff in both training models completed action plans as part of the intervention.

#### Fidelity

Fidelity of intervention delivery refers to the degree to which an intervention is delivered as intended. In this study, we measured fidelity in the in-person training model via a self-report checklist completed by the facilitators directly after each in-person learning collaborative training session. This tool included 5-point ratings for all learning objectives intended to be delivered at each session (“1” ratings indicated “needs improvement or not at all clear” and “5” ratings indicated “excellent or very clear“), as well as sections to report on facilitation (e.g. time dedicated to each objective, organization, and participation), materials distributed, and qualitative feedback on successes and challenges. The online model was delivered with uniform fidelity given its automated, centralized nature and, thus, no fidelity measures were collected or reported.

## Statistical analysis and sample size calculation

We examined the difference between each intervention arm and control sites in change in the primary outcome, the OSNAP-OPAT aggregate healthy practice score, using linear mixed regression models. Each day of practice data provided by each site was included as an observation, and models accounted for repeated observations of days within sites. Denominator degrees of freedom were estimated using the between-within method, and repeated observations within sites were assumed to have a compound symmetry variance-covariance structure. We assessed difference in score change over time using an interaction term indicating the follow-up time period x OSNAP intervention arm. To examine change in secondary outcomes, meeting healthy practice goals for specific behaviors, we fit generalized linear regression models with a logit link and binary distribution, with similar specifications as the primary outcome models. We report the mean percentage of days each goal was met by sites at baseline and follow-up, and odds ratios from the regression models indicate the likelihood of intervention sites meeting the goal on more or less days at follow-up vs. baseline compared with controls. An intention-to-treat analysis was used to assess effectiveness outcomes. Descriptive statistics were calculated for cost, fidelity, and adoption outcomes.

Prior to study initiation, we estimated that with an average of 4 days of practice data per time point obtained from 30 sites (10 each OSNAP in person, OSNAP online, and control) we would have 80% power to detect an increase of one OSNAP goal in each OSNAP arm compared to controls.

## Results

Fifty-three of the 70 (76%) randomized afterschool sites completed baseline and follow-up OSNAP-OPAT data collection and comprise our longitudinal sample for analysis of effectiveness outcomes. Figure 1 depicts site enrollment and loss to follow up in detail. Eighteen of the 24 sites allocated to the in-person arm and 13 of the 23 sites allocated to the online arm received the intervention and completed longitudinal data. Two sites allocated to the in-person arm and one allocated to the online arm completed longitudinal data, but did not participate in the intervention; these sites are included in our intent-to-treat analysis of effectiveness. Some sites participated in the intervention, and thus were included in cost and reach estimates, but did not complete longitudinal data (in-person arm: *n* = 1, online arm: *n* = 4) and were not included in this analysis.

Baseline characteristics of the 53 afterschool sites with longitudinal effectiveness data appear in Table 1. Overall, the sample was racially and ethnically diverse with sites reporting serving 47% White children, 24% Black/African American children, and 16% Hispanic/Latino children. Children at afterschool sites ranged in age from four to 15 years, with the average age of 8 years old. The size of sites varied greatly from enrollment of seven to 117, with an average of 39 children and four afterschool site staff members. There were no significant differences in baseline demographics between the control, in-person, and online training arms. Characteristics of the afterschool site staff appear in Table 2.

**Table 1:** Attributes of afterschool sites in the out-of-school time nutrition and physical activity intervention 2016–2017 scale up, by training arm (*N* = 53)

	In person (*N* = 20)	Online (*N* = 14)	Control (*N* = 19)	*p*-value for difference between 3 arms
Region (N)				0.84
East	5	4	3	
Midwest	10	7	9	
South	5	3	7	
Child Enrollment (Mean, SD)	35.4 (21.5)	43.8 (27.2)	40.8 (29.4)	0.64
Number staff (Mean, SD)^a^	3.8 (1.7)	3.9 (2.1)	3.7 (2.0)	0.92
Age of children served (Mean, SD)^b^	8.1 (0.6)	8.0 (0.6)	8.2 (1.4)	0.75
Race/ethnicity of children served^c^				
White	40.4% (30.0)	53.6% (35.1)	49.8% (33.8)	0.60
Black/African American	33.8% (23.9)	20.4% (26.5)	18.1% (19.0)	0.18
Hispanic/Latino	20.5% (20.0)	11.2% (10.1)	14.9% (12.5)	0.29
Asian	2.9% (4.9)	2.6% (4.7)	3.5% (5.1)	0.88
Other	1.0% (3.2)	1.0% (3.2)	6.2% (16.2)	0.36
OSNAP Goals Met at Baseline (Mean, SD)				
Aggregate healthy practice score^d^	6.03 (0.76)	5.25 (1.09)	6.01 (1.72)	0.16
Percent of Days Meeting OSNAP Goal (Mean, SD)				
Provide all children with at least 30 min of moderate to vigorous physical activity every day	41 (38)	44 (46)	50 (43)	0.82
Offer 20 min of vigorous physical activity (3 times per week)	56 (39)	48 (40)	53 (45)	0.88
Do not serve sugary drinks (SSBs or 100% juice larger than 4 oz)	88 (32)	100 (0)	91 (29)	0.38
Do not allow sugary drinks to be brought in during program time	65 (43)	48 (44)	65 (39)	0.44
Offer water as a drink at snack every day	41 (50)	29 (47)	52 (49)	0.39
Offer a fruit or vegetable option every day at snack	58 (37)	45 (36)	70 (38)	0.17
Limit computer and digital device time to homework or instructional only	99 (6)	100 (0)	86 (29)	0.05
Eliminate broadcast and cable TV and movies	99 (4)	99 (5)	96 (9)	0.41
When serving grains (like bread, crackers, and cereals), serve whole grains	55 (40)	20 (31)	39 (35)	0.03

^a^1 in-person site was missing number of onsite staff

^b^1 in-person, 2 online, and 2 control sites were missing mean age of children

^c^Percent race/ethnicity was estimated as the midpoint of a range and therefore percents for each group do not add up to 100%. 8 in-person, 2 online, and 1 control site were missing race/ethnicity

^d^Number of OSNAP goals met per day, out of 9 possible

**Table 2:** Characteristics of afterschool staff in the Out-of-School Time Nutrition and Physical Activity Intervention 2016–2017 scale up, by training arm (*N* = 51)^a^

	In person (*N* = 19)	Online (*N* = 13)	Control (*N* = 19)
Age (Mean, SD)	30.9 (12.5)	30.0 (13.2)	33.7 (12.8)
Women (N, %)	14 (74%)	8 (61.5%)	14 (77.8%)
Race/ethnicity (%)			
Black or African American	36.8%	15.4%	36.8%
Hispanic or Latino	21.1%	30.8%	15.8%
White	42.1%	53.9%	47.4%
Other	10.5%	7.7%	0.0%
Highest level of education (%)			
High school	15.8%	8.3%	21.1%
Some college/Associates	47.4%	50.0%	57.9%
College	36.8%	33.3%	15.8%
Graduate school or higher	0.0%	8.3%	5.3%
Years of experience (Mean, SD)	4.7 (6.3)	4.2 (2.6)	4.6 (5.6)

^a^Full survey data missing from two longitudinal sites, one control site missing gender data and one online site missing education data

### Primary effectiveness outcomes: afterschool practice change

At baseline, intervention sites participating in the in-person training met 6.03 (SD 0.76) of 9 goals and those participating in the online training met 5.25 (SD 1.09) of 9 goals. Sites randomized to the in-person intervention served more whole grains than other arms at baseline; all other practices were balanced at baseline (see Table 1). Sites were nearly always meeting goals to not serve sugary drinks, limit computer and digital device time to homework or instruction only, and eliminate broadcast and cable TV and movies. Sites less frequently met the other six OSNAP goals on nutrition and physical activity.

Table 3 presents data on the impact of each training model on the overall afterschool practice change as well as each nutrition and physical activity goal. Intervention sites participating in the in-person training had a significantly larger increase in the aggregate afterschool practice score compared with control sites (mean + 0.44; 95% CI 0.02, 0.86; *p* = 0.04), while intervention sites participating in the online training did not show a statistically significant difference in aggregate practice score change compared with controls (mean + 0.28; 95% CI -0.18, 0.73; *p* = 0.24).

**Table 3:** Change in nutrition and physical activity goals met between baseline (fall 2016) and follow up (spring 2017), by training arm (*N* = 53)

	In Person (*N* = 20)	Online (*N* = 14)	Control (*N* = 19)
Primary outcome			
Aggregate healthy practice score^a^			
Baseline Mean (SD)	6.03 (0.76)	5.25 (1.09)	6.01 (1.72)
Follow Up Mean (SD)	6.78 (1.69)	6.01 (1.09)	6.48 (1.51)
Difference in Change vs. Control, Mean (95% CI)^b^	0.44 (0.02, 0.86)	0.28 (−0.18, 0.73)	Ref
*p*-value	0.04	0.24	
Secondary outcomes			
Provide all children with at least 30 min of moderate to vigorous physical activity every day			
Baseline % of Days Meeting Goal, Mean (SD)	41 (38)	44 (46)	50 (43)
Follow Up % of Days Meeting Goal, Mean (SD)	59 (44)	49 (40)	53 (40)
Odds Ratio (95% CI)^c^	1.80 (0.54, 6.00)	1.01 (0.25, 4.08)	Ref
*p*-value	0.34	0.99	Ref
Offer 20 min of vigorous physical activity (3 times per week)			
Baseline % of Days Meeting Goal, Mean (SD)	56 (39)	48 (40)	53 (45)
Follow Up % of Days Meeting Goal, Mean (SD)	64 (45)	45 (43)	61 (38)
Odds Ratio (95% CI)^c^	1.13 (0.37, 3.46)	0.56 (0.21, 1.49)	Ref
*p*-value	0.83	0.25	Ref
Do not serve sugary drinks (SSBs or 100% juice larger than 4 oz)			
Baseline % of Days Meeting Goal, Mean (SD)	88 (32)	100 (0)	91 (29)
Follow Up % of Days Meeting Goal, Mean (SD)	90 (23)	93 (27)	94 (23)
Odds Ratio (95% CI)^c, d^	N/A	N/A	Ref
*p*-value^d^	N/A	N/A	Ref
Do not allow sugary drinks to be brought in during program time			
Baseline % of Days Meeting Goal, Mean (SD)	65 (43)	48 (44)	65 (39)
Follow Up % of Days Meeting Goal, Mean (SD)	69 (41)	49 (46)	92 (22)
Odds Ratio (95% CI)^c^	0.23 (0.05, 1.00)	0.19 (0.05, 0.73)	Ref
*p*-value	0.07	0.03	Ref
Offer water as a drink at snack every day			
Baseline % of Days Meeting Goal, Mean (SD)	41 (50)	29 (47)	52 (49)
Follow Up % of Days Meeting Goal, Mean (SD)	79 (41)	40 (48)	53 (50)
Odds Ratio (95% CI)^c^	5.68 (1.46, 22.07)	1.76 (0.59, 5.28)	Ref
*p*-value	0.02	0.32	Ref
Offer a fruit or vegetable option every day at snack			
Baseline % of Days Meeting Goal, Mean (SD)	58 (37)	45 (36)	70 (38)
Follow Up % of Days Meeting Goal, Mean (SD)	75 (32)	74 (34)	59 (42)
Odds Ratio (95% CI)^c^	2.43 (0.84, 6.99)	5.62 (1.74, 18.13)	Ref
*p*-value	0.11	0.01	Ref
Limit computer and digital device time to homework or instructional only			
Baseline % of Days Meeting Goal, Mean (SD)	99 (6)	100 (0)	86 (29)
Follow Up % of Days Meeting Goal, Mean (SD)	94 (23)	100 (0)	86 (31)
Odds Ratio (95% CI)^c, d^	N/A	N/A	Ref
*p*-value^d^	N/A	N/A	Ref
Eliminate broadcast and cable TV and movies			
Baseline % of Days Meeting Goal, Mean (SD)	99 (4)	99 (5)	96 (9)
Follow Up % of Days Meeting Goal, Mean (SD)	96 (18)	100 (0)	99 (5)
Odds Ratio (95% CI)^c, d^	N/A	N/A	Ref
*p*-value^d^	N/A	N/A	Ref
When serving grains (like bread, crackers, and cereals), serve whole grains			
Baseline % of Days Meeting Goal, Mean (SD)	55 (40)	20 (31)	39 (35)
Follow Up % of Days Meeting Goal, Mean (SD)	63 (41)	56 (41)	51 (42)
Odds Ratio (95% CI)^c^	0.85 (0.30, 2.40)	2.94 (0.72, 12.07)	Ref
*p*-value	0.75	0.13	Ref

Sites completed observations on average 4.0 days per site at baseline and 4.3 days per site at follow up. Baseline and follow up means represent site-level averages

^a^Number of OSNAP goals met per day, out of 9 possible

^b^Difference in change in aggregate healthy practice score between intervention and control arms. Estimated among all observation days within sites, using linear mixed regression models accounting for clustering of days within sites using a compound symmetry variance-covariance matrix and between-within method of estimating denominator degrees of freedom

^c^Odds ratio for difference in change in percent of days meeting goal between intervention and control arms. Estimated among all observation days within sites, using generalized linear regression models with a binomial distribution and logit link, accounting for clustering of days within sites using a compound symmetry variance-covariance matrix

^d^N/A indicates not applicable because models did not converge due to small numbers of sites not meeting goals

Areas of significant improvement were offering water as a drink at snack every day and offering a fruit or vegetable option every day at snack. Improvements in water offerings were significantly different from controls in the in-person training arm (OR 5.68; 95% CI 1.46, 22.07; *p* = 0.02), but not in the online training arm (OR 1.76; 95% CI 0.59, 5.28; *p* = 0.32). Improvements in fruit and vegetable offerings were significantly different from controls in the online training arm (OR 5.62; 95% CI 1.74, 18.13; *p* = 0.01), but not in the in-person training arm (OR 2.43; 95% CI 0.84, 6.99; *p* = 0.11). Change on one goal – not allowing sugary drinks to be brought in during afterschool time – was in the opposite direction as expected. Intervention sites were less likely to increase the percentage of days meeting this goal compared with controls and substantial improvement was observed in the control sites (65% meeting goal at baseline, 92% at follow-up). No significant differences in likelihood of meeting goals from baseline to follow-up in intervention vs. control sites were observed for the other six OSNAP goals.

### Reach

The reach of the intervention varied by training arm (Table 4). A total of 36 afterschool site staff from the 19 sites that received the in person intervention and 19 afterschool site staff from the 17 sites that received the online intervention were trained. On average, 23 afterschool site staff attended in person learning sessions, ranging from 26 at learning collaborative 1, to 24 at learning collaborative 2, and 19 at learning collaborative 3. In contrast, an average of 11 afterschool site staff participated in monthly online learning sessions. Participation was highest in the first two sessions, with unique participant log-ins at 18 in Session 1 and 17 in Session 2, and then dropped to seven or eight unique log-ins during the final three sessions.

**Table 4:** Estimated cost and reach of in-person versus online training models in U.S. dollars, 2016–2017

	In Person	Online
Number of Afterschool Sites Trained	19	17
Number of Afterschool Staff Members Trained	36	19
Total Overall Costs	$24,402	$6383
Cost Breakdown by Activity		
Train the Trainer	$8674	NA
Learning Community Cost	$14,696	$6266
Technical Assistance Cost	$1032	$117
Cost Breakdown by Payer		
Research team	$3819	$1370
Associations	$20,583	$5013
Cost Per Afterschool Staff Member Trained	$678	$336
Cost Per Site Trained	$1284	$375

### Cost

The estimated total cost of the in-person OSNAP training is $24,402 ($678 per person trained, $1284 per site trained) and the estimated total cost of the online training is $6383 ($336 per person trained, $375 per site trained). Details of the training costs appear in Table 4. The largest costs in the in-person implementation strategy were the learning collaborative sessions, which accounted for 60% of the total cost, followed by the 6-h initial training for facilitators, which accounted for 36% of the total cost. For the in-person model, we estimate that our research team accrued 16% of the total implementation cost, and each Association between 24 and 32% of the total cost, ranging from $5952 to $7742 per Association. The estimated cost of the online training was less than the in-person, at only $6383. Ninety-eight percent of the cost was for the online learning collaborative. For the online model, we estimate that our research team accrued 22% of the total implementation cost, and each Association between 20 and 30% of the total cost, ranging from $1287 to $1910 per Association.

### Adoption

**Fig. 3:**
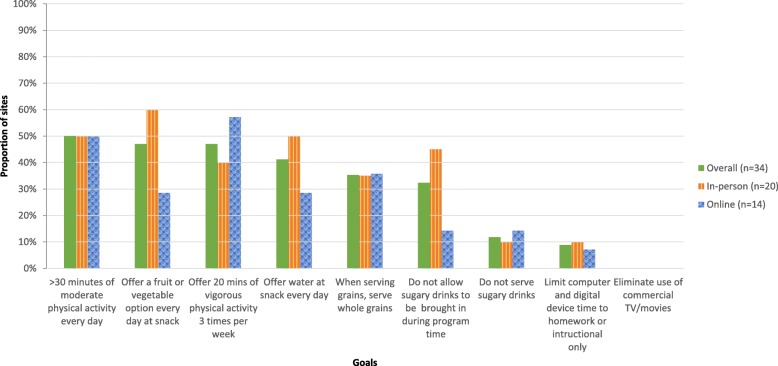
Proportion of intervention sites selecting each OSNAP goal

Figure 3 depicts adoption of the OSNAP goals. Among the 34 intervention sites with complete baseline and follow-up data, the most commonly selected goals were 1) provide greater than 30 min of moderate physical activity every day (50% of sites), 2) offer a fruit or vegetable option every day at snack (47% of sites), 3) offer 20 min of vigorous physical activity three times per week (47% of sites), and 4) offer water at snack every day (41% of sites). Few sites selected screen time goals.

### Fidelity

Facilitator ratings of learning objective delivery across all in-person learning collaborative sessions averaged 4.1 out of a possible score of 5 (excellent). Ratings were highest for session 3 (4.4) and lowest for session 2 (3.6).

## Discussion

This study demonstrates that an evidence-based afterschool intervention can be successfully scaled-up for broad impact and population reach in this pilot trial implemented in multiple sites in three states. The in-person train-the-trainer implementation strategy yielded improvements in the number of targeted nutrition and physical activity practices. Much of this change was driven by improvements in the beverage environment. The online training model showed promising effects for increased offerings of fruits and vegetables and positive trends for improvement, but did not show significant comprehensive changes in the primary outcome. Overall, both training models had low implementation costs, with most of the resources going to staff time. These staffing costs mirror data that indicate over half of school year OST costs are attributed to staff salaries [49]. Given that the average youth serving OST site costs approximately $4320 per slot annually [49], with an average enrollment of 39 children in this study, OSNAP would amount to a small fraction of this operating cost at only $33 per child.

Differences in afterschool site staff reach between the in-person and online intervention arms contributed to total cost differences and may have contributed to differences in effectiveness. Fewer participants per site took part in online trainings so while the online training cost per person trained was half of the in-person training cost per person trained, total costs of the online training arm as implemented in this study were just over one-quarter the total costs of the in-person arm. With respect to effectiveness, it is possible that the lack of a statistically significant increase in the aggregate healthy practice score in the online arm versus controls was partially driven by the relatively low reach in the online group. More research is needed to refine the online training implementation strategy for improved retention and impact. Best practices for online training are still evolving and this study shows promising results given the limited literature on the impact of online training on practice changes in public health settings [35, 50].

These findings indicate the ability for community-based prevention interventions to be spread with limited resources and meaningful effects. Few interventions of this kind have been rigorously studied beyond small, tightly controlled group randomized trials and thus the potential for translating successful interventions into practice is too often squandered. With a sample of 70 afterschool sites across three geographically and racially/ethnically diverse areas of the U.S. and a rigorous randomized design, our findings help to establish the evidence for implementation strategies that can be used to bring these type of complex, multilevel environmental change interventions to scale. The use of a simple validated measure ensured that data collection was both feasible and rigorous. Generalizability for this study is also high because we tested training models delivered as they would be in real world settings and recruited from an existing dissemination partnership—the YMCA, which is the largest provider of private afterschool programming in the United States. Our research team led implementation of this research study, but we envision that the YMCA of the USA would be running these trainings in the future and designed all activities with input from YMCA leaders and afterschool site staff on our advisory group. While we feel confident generalizing to other YMCA afterschool sites, we cannot generalize to other types of afterschool sponsors (e.g. the Boys and Girls Club, city recreation sites), where scale-up strategies may differ. Our intervention design assigning sites in each Association to the three training arms supports generalizability; however, there was the potential for local contamination. For instance, afterschool staff at intervention sites could have informally shared materials or new practice ideas with staff at control sites or Association leaders may have decided to order more fruits and vegetables for afterschool sites centrally. We also had limited power to detect effects in both intervention arms and test for mediation. Future studies with larger samples would be able to test for interaction effects to determine what types of contextual factors (e.g. staffing expertise, enrollment, funding) influence the impact of the intervention on practice changes.

## Conclusions

This study has successfully tested implementation strategies to initiate scale-up of an evidence-based public health intervention within the YMCA, a large community-based organization with the potential for international reach. Building on the evidence for an in-person training approach from our group-randomized trial, we confirmed that a train-the-trainer approach can effectively spread the intervention while building the capacity of local practitioners. The online training model showed promising positive effects at a lower cost and may be a particularly useful option for geographically disbursed sites and those led by staff with competing commitments, where the time for in-person training is challenging. A larger trial with improved strategies to promote interactivity online could have the power to demonstrate significant effects. Hybrid online/in-person models should be explored for the potential of maximizing impact and minimizing cost. Further research is also needed on how prevention interventions such as OSNAP can be sustained within large organizations in the face of changing priorities, funding shifts, and staff turnover. Building evidence-based intervention delivery into existing training systems and supporting with professional development infrastructure are important next steps for long-term change and health impact. In sum, this study highlights the importance of identifying implementation strategies for interventions in the field of public health prevention where even small health-promoting changes can make a difference when spread across a large population [51].

## Data Availability

Please contact the corresponding author, Rebekka Lee by email at rlee@hsph.harvard.edu, for data requests. OSNAP intervention materials are available on our website at www.osnap.org.

## Abbreviations

EBI: Evidence-based intervention
HEPA: Health Eating & Physical Activity
OSNAP: Out-of-School Nutrition and Physical Activity
OSNAP-OPAT: Out-of-School Nutrition and Physical Activity Observation Practice Assessment Tool
OST: Out-of-school time
